# Comparative Assessment of Out-of-Pocket Health Expenditure in Haemodialysis and Peritoneal Dialysis Patients

**DOI:** 10.3390/healthcare13243325

**Published:** 2025-12-18

**Authors:** Suhaila Saad, Nurulhuda Mohd Satar, Roza Hazli Zakaria

**Affiliations:** 1 Centre for Sustainable and Inclusive Development Studies, Faculty of Economics and Management, Universiti Kebangsaan Malaysia (UKM), Bangi 43600, Malaysia; suhailasaad@ukm.edu.my; 2 Department of Economics, Faculty of Business and Economics, Universiti Malaya, Kuala Lumpur 50603, Malaysia; roza@um.edu.my

**Keywords:** out-of-pocket health expenditures, hemodialysis, peritoneal dialysis, chronic kidney disease

## Abstract

**Background:** Dialysis is a life-sustaining treatment for patients with end-stage renal disease (ESRD), but it requires high financial costs due to the need for continuous treatment and the associated expenses of medical supplies, equipment, and related care. **Objective:** This study aims to compare the out-of-pocket (OOP) health expenditure incurred by patients undergoing haemodialysis (HD) and continuous ambulatory peritoneal dialysis (CAPD). **Methods:** The data for this observational cross-sectional study were obtained through a survey at two public hospitals, comprising a sample of 220 ESRD patients. In order to compare the OOP health expenditure between two modalities, the Mann–Whitney U test and the chi-square test were employed. Multiple linear-regression analysis was then used to identify the contributing factors associated with the aforementioned OOP expenses. **Results:** The estimated median monthly OOP health expenditure for HD patients was MYR 388 (interquartile range [IQR: 224–519]), significantly higher than CAPD (MYR 160 [IQR: 100–231]; *p* < 0.001). Our findings confirm that the choice of dialysis modality significantly affects the OOP health expenditures for dialysis patients $\left(MYR\;145.73;\;95\%\;CI:\;75.51–218.15;\;p\;<\;0.05;\;0.001\right).$ Additional determinants of OOP health expenditure identified in this study include the interaction between the modality choice and the distance from home to the dialysis centre (MYR 3.39; 95% CI: 0.27–6.66; *p* < 0.05; 0.022), comorbidity status (MYR 49.51; 95% CI: 9.09–90.77; *p* < 0.05; 0.031), duration of illness (MYR 4.01; 95% CI: 0.71–7.63; *p* < 0.05; 0.041), and household income MYR 67.43 (95% CI: 1.71–134.81; *p* < 0.05; 0.021). **Conclusions:** This study emphasises the need to improve the training and awareness of CAPD to increase its use, as it requires less travel and lowers OOP expenses. In addition, introducing a travel reimbursement scheme is also recommended to reduce the transportation costs for HD patients.

## 1. Introduction

Chronic kidney disease (CKD) is widely recognised as a major global public health issue due to its significant impact on morbidity and mortality. It was ranked the eighth leading cause of death globally, and responsible for 1.4 million deaths in 2019 [1]. According to a recent study, the global median prevalence of CKD was 9.5% [2]. The prevalence of CKD was attributed to the increase in the elderly, along with the adoption of modern lifestyle behaviours such as tobacco use, the excess consumption of alcohol, unhealthy diet intake, and physical inactivity [3,4]. Although the prevalence of CKD has remained stable in several countries, the absolute number of patients has increased due to population growth [5].

The prevalence of CKD in Malaysia increased from 9.07% in 2011 to 15.48% in 2018, indicating a significant upward trend [6]. According to a report published by the Malaysian Dialysis and Transplant Registry in 2018, the number of new patients registered in 2018 was 8431, nearly double the number in 2008. Consistently with this trend, the total number of dialysis patients rose from 19,430 in 2008 to 44,136 in 2018. The Ministry of Health has projected that around 106,000 Malaysians will be diagnosed with kidney failure by 2040, and 30 percent of them are expected to be young adults under the age of 45. The growing prevalence of kidney failure cases is an alarming concern as it will strain the healthcare system while simultaneously imposing substantial financial pressures on patients and their families [7,8].

In Malaysia, dialysis patients who are treated at public healthcare facilities receive substantial government subsidies. This includes the use of dialysis machines for haemodialysis (HD) patients, the purchase of dialysis solutions and consumables for peritoneal dialysis (PD) patients, and the payments of consultation fees and medicines [9]. Treatment costs are fully subsidised for government employees and their dependents [10]. Patients covered by the Social Security Organisation (SOCSO) or religious bodies such as the Zakat institution are eligible to receive reimbursements for some of their medical expenses [11]. For the remaining patients, treatment is provided at a minimal cost.

Apart from the direct costs of dialysis itself, patients are further burdened by expenses related to the management of comorbidities such as diabetes and hypertension [12]. For those undergoing HD, the financial strain is exacerbated by transportation costs, as they are required to travel frequently to dialysis centres several times a week [13]. PD provides the advantage of home administration and eliminates the cost of regular travel to dialysis centres [14]. However, this modality imposes recurring financial demands, particularly for consumables such as dialysis solution bags, catheters, dressings, and ancillary equipment essential for safe treatment [9,15]. Moreover, PD requires a clean environment to reduce the risk of infection, which often necessitates additional household expenditures to maintain proper hygiene standards [16].

Although most of the previous studies have generally indicated that HD is associated with higher financial costs compared with PD [9,15], other studies have found little or no significant difference between the two modalities [9,17]. The inconsistency in findings highlights the need for further investigation, particularly given that most of the previous studies were conducted from the perspective of third-party payers, providers, or healthcare systems. Evidence from patient’s perspective remains limited [18,19,20,21,22,23], and available studies predominantly focus on HD [7,19,22,24,25] with very few offering a direct comparison of OOP expenditure between HD and CAPD. Addressing this gap is crucial in settings where patients are responsible for substantial non-reimbursable expenses. Therefore, this study aims to address this gap by estimating and comparing the OOP health expenditure incurred by HD and CAPD patients, as well as to examine the key cost components and drivers contributing to variations in OOP expenditure.

## 2. Materials and Methods

### 2.1. Study Design

This study employed an observational cross-sectional design, which is commonly used for assessing patient characteristics and healthcare burden [26]. Data was obtained through a survey conducted at two public hospitals in Selangor: Serdang Hospital and Kajang Hospital (Supplementary File S1). Private hospitals were not included because almost 99% of PD patients in Malaysia were treated at public health facilities [9]. Similarly, patients from NGO-run centres were not included, as the level and consistency of financial support provided by these organisations varied considerably.

Patients were given a questionnaire developed to estimate the out-of-pocket (OOP) health expenditure of dialysis patients, and it was completed with minimal guidance from the researcher. This questionnaire was reviewed by three experts to establish content validity, which was to ensure that the items were relevant, clear, and representative of the construct being measured [27]. The survey captured information on direct medical costs, such as dialysis treatment and comorbidity management, as well as direct non-medical costs, particularly transportation. All costs were assessed from the perspective of patients and households. However, it is important to note that all cost data were obtained through patient self-reporting, which introduces the possibility of recall bias. As the sample was drawn from only two public hospitals in Selangor, the findings may not fully represent the broader ESRD population in Malaysia. The survey was conducted between May 2022 and August 2022 and included both HD and continuous ambulatory peritoneal dialysis (CAPD) patients. Ethical approval for this study was obtained from the Medical Research and Ethics Committee (No. 22-00652-WEB (2) on 22 April 2022).

### 2.2. Sampling and Data Collection

The sample size was determined using the G*Power version 3.1.9.7, which calculated the sample size based on information from a similar previous study [9]. A proposed sample size for this study was 220 patients with a minimum of 110 patients per HD and PD group. The sample size was limited as only two of the five targeted hospitals granted approval, and PD services were available in relatively few public hospitals compared with the widespread provision of HD [10].

Systematic random sampling was used to select participants at each hospital. Every fourth patient who arrived for treatment on a given day was selected and screened based on the inclusion criteria. Participants were provided with all relevant information (objectives of the study, procedures, risks, benefits, alternatives to participation, etc.) before being given the participant information sheet (PIS). Participants were then given adequate time to consider whether they were keen to participate in the study and had the opportunity to ask the researcher any additional questions. If the participants were satisfied and willing to participate in the study, they were asked to sign a written agreement (information consent form). Patients would need about 20 min to complete the questionnaire. Those who chose not to participate were excluded from the study and new patients were recruited randomly. All patients received a Malay version of the questionnaire to match their linguistic background and facilitate understanding.

### 2.3. Inclusion and Exclusion Criteria

The recruitment was restricted to eligible patients available during the study period. The inclusion criteria were as follows: (1) adults aged 18 years and above; (2) diagnosed with ESRD and receiving HD or CAPD at the participating hospitals for at least three months; (3) able to communicate effectively and demonstrate adequate cognitive ability to understand the survey questions and provide reliable responses [28]; (4) without serious medical complications that could impair participation; and (5) willing to provide informed consent. Patients were excluded if they (1) were younger than 18 years; (2) had mental disorders, impaired consciousness, or communication difficulties that could compromise their ability to provide reliable responses; (3) had serious medical complications; or (4) declined to participate in the study. They were then replaced by the next subject to enter the hospital.

### 2.4. Variables and Cost Calculation

This study was carried out from the perspective of patients and households. The costs estimated were divided into direct medical costs and direct non-medical costs. Direct medical costs encompassed the expenses related to dialysis treatment, covering the costs of HD machines, dialysis solutions, consumables, and medications. The consumables included face masks, dressings, adhesive tapes, and other items used to maintain a sterile environment during the dialysis procedure. The costs related to comorbidity management were also incorporated under the direct medical costs, while direct non-medical costs were estimated based on the expenditure on transportation. However, certain expenses, such as hospitalisation costs, were not included because many of the patients were unable to recall the expenses related to their hospital stays. OOP expenditure was assessed using a one-month recall period. Patients were asked to report the total amount they spent on each cost component during the previous month, including their monthly spending on medications and consumables. The OOP health expenditure was calculated based solely on the amounts paid by the patients from their own funds, excluding any reimbursements received.

In addition to cost data, we collected demographic, socioeconomic, and medical-related information to further support and contextualise our findings. Table 1 provides detailed descriptions of all variables included in the study. The socioeconomic variables presented in Table 1 were incorporated as covariates in the analysis of OOP expenditure to evaluate their influence on patients’ overall OOP health spending.

### 2.5. Statistical Analysis

Analysis began with a baseline comparison among HD and CAPD patients using a descriptive analysis that summarised the main variable, providing an initial overview of group differences before conducting inferential analyses. The calculation of mean, median, and standard deviation was performed besides the calculation of frequency and percentage. Categorical variables were measured and expressed in terms of number and percentage, while the mean (±SD, standard deviation) and median (IQR, interquartile range) were used for continuous variables. The result of the OOP health expenditure was calculated in Malaysian ringgit (MYR).

In the next stage, the Mann–Whitney U test was used to compare the differences in the OOP expenditures between HD and CAPD patients. This test was appropriate for assessing differences in a continuous variable across two independent categorical groups. The chi-square test of independence was used then to compare categorical demographic and socioeconomic characteristics between HD and CAPD patients. This test is suitable for examining relationships between categorical variables and determining whether the observed frequencies differ significantly from what would be expected by chance across the two groups.

In addition, multiple linear-regression analysis was conducted to identify the key drivers and contributing factors of OOP health expenditure among dialysis patients. This method is appropriate for modelling a continuous dependent variable and allows the assessment of the independent effect of each predictor on OOP health spending. By adjusting for relevant covariates, including sociodemographic and clinical characteristics as well as dialysis modality uptake, the model provides a clearer understanding of the factors that significantly influence variations in OOP expenditure. The multiple linear-regression model for OOP health expenditure is presented in Equation (1).

(1) $${OOP}_{HE}=f(Modality,\;Distance,\;Modality\;*\;Distance,\;Comorbidity,\;DurationIllness,\;Gender,\;Elderly,\;LHHIncome,\;FulllySub)$$

The differences between the groups, as well as the independent variable, were assessed based on a two-sided *p*-value threshold of 0.05. All analyses in this study were conducted using the Statistical Package for Social Sciences (SPSS), version 26.

### 2.6. Subgroup Analysis

The baseline model is developed using data from all patients to estimate the factors influencing OOP health expenditure in dialysis patients. However, it is argued that the cost of dialysis fluctuates during the initial stages [29]. Therefore, to ensure the robustness of the analysis, a subgroup analysis was conducted by excluding patients with a duration of illness of one year or less.

## 3. Results

According to Table 2, the mean (±SD) age of the study population was 48.4 (±14.7) years. Almost half of the patients were 50 years old and above and the number of female patients was slightly higher than the male counterparts. In terms of education, more than half of the households were found to have at least one member who studied up to tertiary education. In terms of economic background, it shows that 61.8% of patients were from the bottom 40% income household group and 41.8% of patients were household heads. Apart from receiving full exemption under the government subsidy programme, some other patients financed treatments entirely using OOP. Other sources are from SOCSO and Zakat, a religious body that actively participates in channelling assistance to dialysis patients. Data concerning medical background showed that the prevalence of both comorbidities, hypertension (71.8%) and diabetes mellitus (45.9%), were high among patients. The result of the independent chi-square test found that HD and CAPD patients differed significantly in terms of household heads (*p* < 0.05; 0.014) and comorbidities (<0.05; 0.000).

### 3.1. Out-of-Pocket Health Expenditure

According to Table 3, the average monthly OOP expenditure of dialysis patients is MYR 282.07 (±183.47) with transportation expenses (MYR 153.50 ± 164.88) slightly exceeding the medical expenses (MYR 128.57 ± 106.07). At least half of HD patients spent MYR 388.00 (IQR: 224.25–519.25) per month, which was significantly higher than the median monthly OOP health expenditure of CAPD patients at MYR 160.00 (IQR: 100.00–231.25) (*p* < 0.05; 0.006). This difference is attributable to transportation expenditure, which was substantially higher among HD patients (MYR 245.00, IQR: 150.00–360.00) compared with CAPD patients (MYR 25.00, IQR: 10.00–50.00), a difference that was statistically significant (*p* < 0.05; 0.000).

### 3.2. Factors Influencing Out-of-Pocket Health Expenditure in Dialysis Patients

The results of multiple linear regression, as shown in Table 4, indicate significant differences in OOP health expenditures between the two dialysis modalities. HD patients experienced higher OOP health expenditure (MYR 145.73; 95% CI: 75.51–218.15; p < 0.05; 0.001)  compared with those undergoing CAPD. In addition, OOP health expenditure is also influenced by the interaction between dialysis modality and distance to the dialysis centre. For each additional kilometre of distance, the OOP health expenditure for HD patients increases by MYR 3.39 (95% CI: 0.27–6.66; p < 0.05; 0.022) more than for PD patients. Dialysis patients with comorbidities incur significantly higher OOP health expenditures with an additional MYR 49.51 (95% CI: 9.09–90.77; p < 0.05; 0.031)  per month compared with those without comorbidities. Furthermore, the duration of each additional year of illness is associated with an MYR 4.01 (95% CI: 0.71–7.63; p < 0.05; 0.041) increase in OOP health expenditure. The findings further reveal that a one percent increase in the household income will potentially increase the OOP health expenditure by MYR 67.43 (95% CI: 1.71–134.81; p < 0.05; 0.021)  per month.

### 3.3. Result of Subgroup Analysis

Table 5 reports the results of multiple linear regression for the subgroup analysis. Patients with an illness duration of less than one year were excluded, as the early phase of dialysis treatment was characterised by elevated and unstable costs that did not reflect long-term expenditure patterns [29]. After adjusting for the duration of illness, the dialysis modality continues to be a significant determinant of the total OOP health expenditure, with HD patients incurring higher OOP health expenditure (MYR 145.97; 95% CI: 67.56–234.12; p < 0.05; 0.003)  than CAPD patients. For each additional kilometre of distance, the OOP health expenditure for HD patients increases by MYR 4.91 (95% CI: 1.89–7.87; p < 0.05; 0.003) more than for PD patients. Household income was also significantly associated with total OOP health expenditure, with an estimated increase of MYR 89.05 (95% CI: 15.93–166.27; p < 0.05; 0.014). The result also indicated that fully subsidised patients had significantly lower OOP health expenditure  (MYR 63.44; 95% CI: −122.45–1.63; p < 0.05; 0.035)  compared with other patients. All other variables demonstrated similar results except for comorbidity status, which was no longer significant in contrast to the baseline model.

## 4. Discussion

Overall, the estimated monthly mean of OOP health expenditure for dialysis patients was MYR 282.07 (USD 60.66). The estimated result was lower than many of the previous studies, including in Indonesia (USD 653.8), Nepal (USD 269.26), Sudan (USD 321.59), and the United States (USD 442) [29,30,31,32]. The OOP health expenditure for dialysis in Malaysia is lower than many other countries, due to the substantial government subsidies provided through public healthcare facilities and also financial support programmes under government and non-government agencies including the SOCSO, Zakat organisation, and National Kidney Foundation [10].

The difference in dialysis expenses between Malaysia and other countries is primarily due to variations in government funding and insurance coverage. Although Nepal and Indonesia provide free or heavily subsidised dialysis treatment under public or national health schemes, OOP health expenditure remains substantial for many patients because the coverage is not comprehensive. For example, the health insurance scheme in Nepal provides free HD sessions in designated hospitals; however, the limited coverage often results in additional OOP health expenditure for patients. These include costs for medications, transportation, diagnostic investigations, and other related medical costs [33]. Similarly to Indonesia, although dialysis coverage is offered under Indonesia’s National Health Insurance scheme, patients are still required to pay monthly premiums that vary by income category [34]. The government subsidises premiums only for the lowest-income groups, whereas others must pay according to their income level, which may restrict access for some patients. In Sudan, insufficient government funding and the lack of facilities in the public sector compel patients to seek treatment at private sectors, which is often expensive and burdensome [35]. In a high-income country such as the United States, dialysis treatment is covered under both public and private insurance schemes but still imposes significant OOP health expenditure. This is due to the insufficient coverage of public insurance such as the Medicare programme that does not include all associated costs. The available private insurance often mandates co-payments and applies substantial deductibles, resulting in considerable OOP health spending in patients.

The relatively low OOP health expenditure among dialysis patients in Malaysia may not be sustainable. This is due to the growing number of patients who exert pressure on the government’s fiscal capacity, whereby maintaining subsidies becomes increasingly difficult for the government. As the findings indicate that CAPD incurs lower costs, increasing PD uptake among clinically suitable patients may be a consideration. This could involve strengthening facilities, improving healthcare worker training, and enhancing the awareness of PD as a treatment option. This strategy has been adopted in countries such as Denmark, France, the United Kingdom, Thailand, Canada, and Hong Kong [36,37]. Specifically, the ratios were 1.94 (United Kingdom), 1.39 (France), 1.90 (Canada), 1.34 (Denmark), 1.58 (New Zealand), and 2.35 (Hong Kong) [38], which indicates that PD is generally cheaper than HD. Furthermore, the government also recommended developing a risk-pooling mechanism with the purpose of distributing financial risks across a larger population for alleviating financial burden. A combination of public and private funding will establish more efficient mechanisms that will ensure the sustainability of the system.

The result of the chi-square test demonstrates that the average monthly OOP health expenditure for HD patients (MYR 385.43 ± 187.11) is two times significantly higher than CAPD patients (MYR 178.72 ± 105.18; *p* < 0.05: 0.000). The findings are aligned with the previous studies, which highlight that HD utilisation is costlier than PD. Prior studies reported a similar result emphasising that the OOP health expenditure for HD patients was over two times more than PD in Thailand [39]. Likewise, the costs for HD uptake were slightly more expensive than for CAPD in Taiwan [40]. The annual cost ratio of HD to CAPD was reported to be 1.17 in Southern China [41].

The result of multiple linear regression confirmed this finding by emphasising the fact that the total OOP health expenditure of HD patients was higher at MYR 145.97 (95% CI: 67.56–234.12; *p* < 0.05; 0.003) per month compared with CAPD patients. A similar result was reported in a restricted model in which HD utilisation incurred higher expenses than CAPD. Transportation was identified as a major contributor to OOP health expenditure, largely due to the substantial travel requirements associated with HD. This was consistent with other studies [40], which emphasised that the main differences in OOP health expenditure between HD and PD patients were due to the transportation expenses. HD patients incur higher transportation expenditure primarily due to the thrice-weekly treatment schedule compared with PD patients who generally need to make only monthly follow-up visits. In addition to these frequent visits, the transportation burden is also influenced by several contributing factors. A key driver is travelling distance, evidenced by the significant interaction between dialysis modality and distance to the treatment centre identified in this study. Socioeconomic conditions further influence the patients’ transportation choice, whereby, lower income often rely on family members or private vehicles and may lack access to affordable alternatives such as public transport [42].

The availability and suitability of public transportation remain major challenges. The lack of direct connections to healthcare facilities and inability of public transport services to accommodate patients’ physical and medical needs make public transport impractical for many patients. Our findings support this as 79.1% of respondents reported feeling safer and more comfortable using their own vehicle when travelling for treatment. This preference suggests that patients choose private vehicles not only due to safety considerations but also because of the structural limitations of public transportation in meeting the specific needs of dialysis patients.

The results underscore the necessity of expanding financial aid to encompass transportation-related expenses such as a travel reimbursement scheme that will provide financial assistance or reimbursement for dialysis patients. A similar programme has been implemented in several countries including the United Kingdom and Canada. The Healthcare Travel Costs scheme in England was introduced to reduce transportation expenses for patients by reimbursing travel costs, while non-emergency patient transport services provided additional transportation support specifically for dialysis patients [43]. Likewise, some programmes in Canada were designed to reduce transportation costs for dialysis patients. For example, Ontario offers reimbursement for out-of-country dialysis treatments through its Out-of-Country Haemodialysis Reimbursement programme, and British Columbia provides travel assistance through its Travel Assistance programme and related travel-loan support. These travel-assistance initiatives do not only reduce the financial burden of long-distance journeys for dialysis patients, but they are also able to improve the access to care at lower OOP costs. Such an initiative may have potential applicability in Malaysia, especially one which is tailored to align with Malaysia’s available resources as well as socioeconomic and geographical factors.

Promoting PD uptake among clinically suitable patients may be another feasible option, given the comparatively lower costs of CAPD as a home-based treatment. The significant differences in cost between the use of HD and PD have led many countries to actively promote PD uptake. This is primarily due to the fact that PD is a more economical alternative and does not involve significant resource consumption as compared with HD [36]. This recommendation aligns with evidence from Taiwan and Thailand, where national policies have successfully promoted PD utilisation as part of their dialysis strategies. Government efforts to initiate a similar policy could help widely promote the use of PD, especially among new patients in the future.

Targeted financial assistance is particularly important, as certain groups bear disproportionately higher OOP health expenditure. Lower-income patients experience significantly greater financial strain compared with higher-income groups, highlighting the need for additional support. Patients with multiple comorbidities may also require enhanced financial protection, given their higher overall healthcare expenditures, especially for hospitalisation. Furthermore, individuals with a longer duration of illness may benefit from long-term assistance mechanisms rather than short-term support, as their OOP health expenditure tend to accumulate over time. Collectively, these evidence-based considerations can help inform more equitable and sustainable dialysis financing strategies within the Malaysian healthcare context.

However, several limitations should be noted when interpreting these findings. First, data were collected from only two public hospitals in Selangor, which limited the generalisability of the findings to the broader ESRD population in Malaysia. Second, the study was conducted within the Malaysian healthcare context, where subsidy structures, dialysis practices, and cost components might have varied from those in other countries. Therefore, the results may not be fully applicable to international populations. Third, the study focuses specifically on patients with CKD undergoing haemodialysis or peritoneal dialysis, and the findings may not extend to other CKD stages or non-dialysis populations. Fourth, certain cost components were not captured in this study, particularly expenses related to hospitalisation. These costs may be significant, especially for vulnerable groups who are more prone to complications, such as patients with multiple comorbidities and older adults. The main reason for excluding hospitalisation costs was the difficulty in accurately determining the charges incurred during inpatient stays. Hospitalisation fees varied according to the type and severity of complications, and many patients were unable to recall the associated expenses reliably.

Despite these limitations, the study remains valuable. By narrowing the focus to one type of healthcare setting (public centre), the study can better control the confounding variables and produce more consistent cost estimates. This approach offers valuable insights into the actual costs borne by dialysis patients within Malaysia’s public healthcare system while also providing useful information for policymakers.

## 5. Conclusions

Dialysis is a high-cost treatment, and the choice of dialysis modality plays a crucial role in determining the financial burden on patients. HD typically incurs higher costs than CAPD, primarily due to the significant transportation expenditure, as HD utilisation requires frequent trips to a dialysis centre. On the other hand, as a home-based treatment, CAPD has relatively lower transportation expenditure, resulting in reduced financial burden for patients. Our findings suggest that reducing the financial burden on patients may require coverage arrangements that include transportation reimbursement and support from a sustainable financing mechanism, such as risk-pooling through social health insurance. Although the selection of dialysis modality should primarily be based on the patient’s medical condition, providing patients with clear information on the costs associated with each treatment option could help support more informed decision-making.

## Figures and Tables

**Table 1:** Variables descriptions.

Variable Name	Abbreviation	Description
Total out-of-pocket	OOP	The amount of money spent by the patient in a month to cover medical expenses and transportation costs during the period of seeking treatment
Total medical expenses	MedicalExp	The total monthly expenses incurred by the patient for medical purposes, i.e., costs related to dialysis treatment such as payment for the use of HD machines, purchase of PD solution, consumable items, and diet intake such as expenses on healthy foods and supplements; this cost does not include inpatients costs
Total dialysis expenditure	DialysisExp	Total monthly expenditure incurred for dialysis treatment only
Total additional medical expenditure	Add.Medical	Total additional monthly medical expenditure
Total transportation expenditure	TransportExp	The monthly expenditure allocated for transportation to the hospital for medical treatment
Explanatory Variables		
Demographic and socioeconomic		
Age	Elderly	Continuous variable
		Aged 18 years and above
		Categorical variables
		1 if patient is 50 years old and above
		0 if patient is 18 to 49 years old
Gender	Gender	1 if patient is a male
		0 if patient is a female
Education level	EducLevel	1 if at least one person in the household has tertiary education level.
		0 if there is no member in the household achieved tertiary education.
Household income category		Continuous variable
	LHH Income	The income variable that has been transformed using the natural logarithm (log transformation)
		Continuous variable
	B40	The bottom 40% of income earners
	M40	The middle 40% of income earners
	T20	The top 20% of income earners
Household head	Breadwinner	1 if the patient is a family breadwinner
		0 if the patient is not the family breadwinner
Distance	Distance	The distance from the residential area to the dialysis centre.
Funding sources		Categorical variables
	Government Exemption	Full government exemption received by civil servants, pensioners, or their dependents, as well as unfortunate patients under the Department of Social Welfare, including the poor and the disabled
	SOCSO	Partially subsidised and the remaining cost covered by social workers insurance fund, which is funded through a collective contribution from both employees and employers in the private sector
	Zakat/Baitulmal	Partially subsidised and the remaining covered by zakat, a religious body
	NGO	Partially subsidised and the remaining covered by other sources such as NGOs or employer companies
		Binary variable
	Fullysub	1 if the patient received full exemption by the government
		0 if the patient does not receive full subsidy
Medical-related variables		
Comorbidity		1 if the patient has diabetes mellitus as a comorbidity
		0 if the patient does not have diabetes mellitus as a comorbidity
Duration of Illness		Number of years suffering from illness
Modality Selection		1 if the patient is receiving haemodialysis treatment (HD)
		0 if the patient is receiving continuous ambulatory peritoneal dialysis (CAPD)

**Table 2:** General characteristics of dialysis patients.

Variable	Total	HD	CAPD	χ^2^	Sig.
	(n = 220)	(n = 110)	(n = 110)		
Age, n (%)				2.92	0.404
Mean (SD)	48.4 (14.7)	46.9 (15.1)	49.8 (14.1)		
Median (IQR)	48.0 (37.0–60.8)	48.5 (35.0–58.3)	48.0 (39.8–62)		
>50	125 (56.8)	64 (58.2)	61 (55.5)		
50–59	37 (16.8)	20 (18.2)	17 (15.5)		
60–69	39 (17.7)	20 (18.2)	19 (17.3)		
≥70	19 (8.6)	6 (5.5)	13 (11.8)		
Gender, n (%)				0	1
Male	96 (43.6)	48 (43.6)	48 (43.6)		
Female	124 (56.4)	62 (56.4)	62 (56.4)		
Education level, n (%)				2.304	0.512
No schooling	1 (0.5)	1 (0.9)	0 (0)		
Primary	8 (3.6)	5 (4.5)	3 (2.7)		
Secondary	75 (34.1)	40 (36.4)	35 (31.8)		
College/University	136 (61.8)	64 (58.2)	72 (65.5)		
Income category, n (%)				0.321	0.852
B40	136 (61.8)	70 (63.6)	66 (60.0)		
M40	71 (32.3)	34 (30.9)	37 (33.6)		
T20	13 (5.9)	6 (5.5)	7 (6.4)		
Household head, n (%)	92 (41.8)	37 (33.6)	55 (50.0)	6.053	0.014 *
Source of funding, n (%)				10.673	0.058
Government exemption	67 (30.5)	36 (32.7)	31 (28.2)		
Out-of-Pocket, OOP	66 (30.0)	35 (31.8)	31 (28.2)		
SOCSO	50 (22.7)	18 (16.4)	32 (29.1)		
Zakat/Baitulmal	25 (11.4)	11 (10.0)	14 (12.7)		
NGOs	9 (4.1)	7 (6.4)	2 (1.8)		
Others	3 (1.4)	3 (2.7)	0 (0.0)		
Comorbidities, n (%)				19.933	0.000 *
Diabetes Mellitus	101 (45.9)	34 (30.9)	67 (60.9)		
Hypertension	158 (71.8)	68 (61.8)	90 (81.8)		
Transportation Mode					
Own/family car	174 (79.1)	83 (75.5)	91 (82.7)		0.191
Bus	2 (0.9)	2 (1.8)	0 (0.0)		
E-hailing (Grab/Taxi)	42 (19.1)	23 (20.9)	19 (17.3)		
Others	2 (0.9)	2 (1.8)	0 (0.0)		

All categorical variables were analysed by Pearson χ^2^ test; significance value * *p* < 0.05; HD, haemodialysis; CAPD, continuous ambulatory peritoneal dialysis; IQR, interquartile range; SD, standard deviation.

**Table 3:** Out-of-pocket health expenditure in dialysis patients.

Variables	Total		HD		CAPD		U (Sig)
	Mean (±SD)	Median (IQR)	Mean (±SD)	Median (IQR)	Mean (±SD)	Median (IQR)	
OOP	282.07 ± 183.47	230.00 (130.00–401.50)	385.43 ± 187.11	388.00 (224.25–519.25)	178.72 ± 105.18	160.00 (100.00–231.25)	2106.5 (0.000) *
Medical Expenditure	128.57 ± 106.07	100.00 (30.00–200.00)	112.56 ± 109.74	62.50 (24.75–187.00)	144.57 ± 100.22	112.50 (63.00–200.00)	7334.5 (0.006) *
Dialysis Expenditure	106.08 ± 91.77	100.00 (30.00–156.00)	84.72 ± 88.71	50.00 (17.50–156.00)	127.44 ± 90.18	100.00 (50.00–200.00)	7842.5 (0.000) *
Additional Medical Expenditure	22.49 ± 62.63	0.00 (0.00–0.00)	27.85 ± 69.88	0.00 (0.00–0.00)	17.14 ± 54.21	0.00(0.00–0.00)	5660.0 (0.205)
Transportation Expenditure	153.50 ± 164.88	80.00 (20.00–247.50)	272.86 ± 158.73	245.00 (150.00–360.00)	34.15 ± 28.56	25.00 (10.00–50.00)	501.5 (0.000) *

All continuous variables were analysed by Mann–Whitney U test; All values are reported in MYR, Malaysian ringgit; HD, haemodialysis; CAPD, continuous ambulatory peritoneal dialysis; OOP, out-of-pocket expenditure; IQR, interquartile range; SD, standard deviation; U, Mann–Whitney U statistic; * *p* < 0.05.

**Table 4:** Factors associated with out-of-pocket health expenditure in dialysis patients.

Variables	$\beta_{i}$	*t*	Baseline Model 95% CI			Bootstrap-Adjusted Model 95% CI		
			Sig.	LB	UB	Sig.	LB	UB
Constant	−72.24	−0.169	0.537	−302.27	157.8	0.489	−256.39	100.52
Modality	145.73	4.206	0.000 *	77.43	214.03	0.001 *	75.51	218.15
Distance	−0.94	−1.007	0.317	−2.8	0.91	0.17	−2.47	0.15
ModeXDistance	3.39	2.3	0.022 *	0.49	6.29	0.022 *	0.27	6.66
Comorbidity	49.51	2.177	0.031 *	4.68	94.33	0.031 *	9.09	90.77
Duration of Illness	4.01	2.106	0.036 *	0.26	7.76	0.041 *	0.71	7.63
Gender	−37.12	−1.861	0.064	−76.44	2.21	0.058	−73.8	5.86
Elderly	25.77	1.204	0.23	−16.43	67.98	0.23	−18.69	72.69
LHHIncome	67.43	2.124	0.035 *	4.86	130	0.021 *	1.17	134.81
Fully Subsidised	−41.86	−1.794	0.074	−87.84	4.13	0.07	−84.42	2.19
Adjusted R square, ${\bar{R}}^{2}$			0.369					
F-test (*p*-value)			15.251 (0.000)					

The result of the multivariate regression analysis is in MYR (Malaysian ringgit) for all dialysis patients; CI, confidence interval; *βᵢ*, regression coefficient; *t*, t-statistic; LB, lower bound; UB, upper bound; Sig., significance level; * *p* < 0.05.

**Table 5:** Subgroup analysis (patients with duration illness >1 year).

Variables	$\beta_{i}$	*t*	Baseline Model 95% CI			Bootstrap-Adjusted Model 95% CI		
			Sig.	LB	UB	Sig.	LB	UB
Constant	−119.67	−0.856	0.393	−395.66	156.32	0.339	−374.29	106.56
Modality	145.97	3.272	0.001 *	57.88	234.06	0.003 *	67.56	234.12
Distance	−1.35	−0.919	0.359	−4.26	1.56	0.132	−3.25	1.05
ModeXDistance	4.91	2.508	0.013 *	1.04	8.78	0.003 *	1.89	7.87
Comorbidity	31.33	1.167	0.245	−21.67	84.33	0.256	−24.35	89.2
Gender	−37.76	−1.554	0.122	−85.74	10.23	0.138	−86.06	9.31
Elderly	38.71	1.54	0.126	−10.94	88.36	0.145	−9.09	90.35
LHHIncome	89.05	2.295	0.023 *	12.42	165.68	0.014 *	15.93	166.27
Fully Subsidised	−63.44	−2.162	0.032 *	−121.4	−5.49	0.035 *	−122.45	1.63
Adjusted R square, ${\bar{R}}^{2}$			0.391					
F-test (*p*-value)			12.993 (0.000)					

The result of the multivariate regression analysis is in MYR (Malaysian ringgit); CI, confidence interval; *βᵢ*, regression coefficient; *t*, t-statistic; LB, lower bound; UB, upper bound; Sig., significance level; * *p* < 0.05.

## Data Availability

The original contributions presented in this study are included in the article/Supplementary Materials. Further inquiries can be directed to the corresponding author.

## Supplementary Materials

The following supporting information can be downloaded at https://www.mdpi.com/article/10.3390/healthcare13243325/s1, File S1: Data set.

## Abbreviations

The following abbreviations are used in this manuscript:

CKD	Chronic Kidney Disease
HD	Haemodialysis
PD	Peritoneal Dialysis
CAPD	Continuous Ambulatory Peritoneal Dialysis
OOP	Out of pocket
SOCSO	Social Security Organisation

## References

[1] Raferty Q., Stafford L.K., Vos T. (2025). Global, regional, and national prevalence of kidney failure with replacement therapy and associated aetiologies, 1990–2023: A systematic analysis for the Global Burden of Disease Study 2023. Lancet.

[2] Jadoul M., Aoun M., Masimango Imani M. (2024). The major global burden of chronic kidney disease. Lancet Glob. Health.

[3] Lee S.J., Chung C.W. (2014). Health behaviors and risk factors associated with chronic kidney disease in Korean patients with diabetes: The fourth Korean national health and nutritional examination survey. Asian Nurs. Res. (Korean Soc. Nurs. Sci.).

[4] Alfano G., Perrone R., Fontana F., Ligabue G., Giovanella S., Ferrari A., Gregorini M., Cappelli G., Magistroni R., Donati G. (2022). Rethinking Chronic Kidney Disease in the Aging Population. Life.

[5] Kovesdy C.P. (2022). Epidemiology of chronic kidney disease: An update 2022. Kidney Int. Suppl..

[6] Saminathan T.A., Hooi L.S., Mohd Yusoff M.F., Ong L.M., Bavanandan S., Rodzlan Hasani W.S., Tan E.Z.Z., Wong I., Rifin H.M., Robert T.G. (2020). Prevalence of chronic kidney disease and its associated factors in Malaysia; Findings from a nationwide population-based cross-sectional study. BMC Nephrol..

[7] Fadzli N.F.A.M., Rasani A.A.M., Keng S.L. (2021). Assessing the financial burden of hemodialysis treatment in Malaysia. Belitung Nurs. J..

[8] Ismail H., Abdul Manaf M.R., Abdul Gafor A.H., Mohamad Zaher Z.M., Ibrahim A.I.N. (2019). Economic Burden of ESRD to the Malaysian Health Care System. Kidney Int. Rep..

[9] Surendra N.K., Manaf M.R.A., Seong H.L., Bavanandan S., Nor F.S.M., Khan S.S.F., Meng O.L., Gafor A.H.A. (2018). The cost of dialysis in Malaysia: Haemodialysis and continuous ambulatory peritoneal dialysis. Malaysian J. Public Health Med..

[10] Bavanandan S., Hooi L.S., Ong L.M., Choo C.L. (2023). 31st Report of the Malaysian Dialysis & Transplant Registry.

[11] Nashruddin S.N.H.M., Wahid H. Pembiayaan Rawatan Pesakit Dialisis Melalui Dana Zakat: Kajian Terhadap Asnaf Al- Gharimin Perubatan Tajaan Lembaga Zakat Selangor. Proceedings of the Persidangan Kebangsaan Ekonomi Malaysia ke-13.

[12] Jie W., Yao M., Wang M., Wang Y., Jia Y., Liu Y., Zou K., Sun X. (2024). Analysis of the Economic Burden of Chronic Kidney Disease with Comorbidities Among Patients in Xuzhou, China. Int. J. Public Health.

[13] Lewis R.A., Bohm C., Fraser F., Fraser R., Woytkiw L., Jurgutis S., Rubin M., Smith G., Buenafe J., Verdin N. (2023). Transportation Burden Associated with Hemodialysis in Canada: A Qualitative Study of Stakeholders. Kidney Med..

[14] Barbado G., Garí C., Ariznavarreta A., Vidal-Vilar N., Alvarez C. (2025). Budgetary impact of increasing use of peritoneal dialysis over haemodialysis in Spain. Health Econ. Rev..

[15] Mushi L., Marschall P., Fleßa S. (2015). The cost of dialysis in low and middle-income countries: A systematic review. BMC Health Serv. Res..

[16] Gursu M., Shehaj L., Elcioglu O.C., Kazancioglu R. (2023). The optimization of peritoneal dialysis training in long-term. Front. Nephrol..

[17] Lin E., Lung K.I., Chertow G.M., Bhattacharya J., Lakdawalla D. (2021). Challenging Assumptions of Outcomes and Costs Comparing Peritoneal and Hemodialysis. Value Health.

[18] Moalosi K., Sibanda M., Kurdi A., Godman B., Matlala M. (2023). Estimated indirect costs of haemodialysis versus peritoneal dialysis from a patients’ perspective at an Academic Hospital in Pretoria, South Africa. BMC Health Serv. Res..

[19] Agada-Amade Y.A., Ogbuabor D.C., Eboreime E., Onwujekwe O.E. (2023). Cost analysis of the management of end-stage renal disease patients in Abuja, Nigeria. Cost Eff. Resour. Alloc..

[20] Bradshaw C., Gracious N., Narayanan R., Narayanan S., Safeer M., Nair G.M., Murlidharan P., Sundaresan A., Retnaraj Santhi S., Prabhakaran D. (2019). Paying for Hemodialysis in Kerala, India: A Description of Household Financial Hardship in the Context of Medical Subsidy. Kidney Int. Rep..

[21] Kaur G., Prinja S., Ramachandran R., Malhotra P., Gupta K.L., Jha V. (2018). Cost of hemodialysis in a public sector tertiary hospital of India. Clin. Kidney J..

[22] Senanayake S.J., Gunawardena N.S., Palihawadana P., Bandara S., Bandara P., Ranasinghe A.U., Karunarathna R.H., Kumara G.P. (2017). Out-of-pocket expenditure in accessing healthcare services among Chronic Kidney Disease patients in Anuradhapura District. Ceylon Med. J..

[23] Gummidi B., John O., John R., Chatterjee S., Jha A., Ghosh A., Jha V. (2022). Catastrophic Health Expenditure and Distress Financing Among Patients with Nondialysis Chronic Kidney Disease in Uddanam, India. Kidney Int. Rep..

[24] Chuengsaman P., Kasemsup V. (2017). PD First Policy: Thailand’s Response to the Challenge of Meeting the Needs of Patients with End-Stage Renal Disease. Semin. Nephrol..

[25] Avirneni H.T., Nandi P., Pawar S.J. (2021). Economic Burden of CKD Among the Beneficiaries of a State-Run Insurance Scheme. Online J. Health Allied Sci..

[26] Vandenbroucke J.P., Von Elm E., Altman D.G., Gøtzsche P.C., Mulrow C.D., Pocock S.J., Poole C., Schlesselman J.J., Egger M. (2014). Strengthening the Reporting of Observational Studies in Epidemiology (STROBE): Explanation and elaboration. PLoS Med..

[27] Yusoff M.S.B. (2019). ABC of Content Validation and Content Validity Index Calculation. Educ. Med. J..

[28] Folstein M.F., Folstein S.E., McHugh P.R. (1975). “Mini-Mental State” A Practical Method for Grading The Cognitive State of Patients for the Clinician. J. Psychiatr. Res..

[29] League R.J., Eliason P., McDevitt R.C., Roberts J.W., Wong H. (2022). Assessment of Spending for Patients Initiating Dialysis Care. JAMA Netw. Open.

[30] Agrawaal K.K., Shah S. (2022). Out of Pocket Expenditure in Patients on Maintenance Hemodialysis: A Single Center Study. Nepal. Med. J..

[31] Younis M., Jabr S., Al-Khatib A., Forgione D., Hartmann M., Kisa A. (2015). A cost analysis of kidney replacement therapy options in Palestine. J. Health Care Organ. Provis. Financ..

[32] Kristina S.A.R.I., Endarti D.W.I., Andayani T.R.I.M., Aditama H. (2021). Cost of illness of hemodialysis in Indonesia: A suvrvey from eight hospitals in Indonesia. Int. J. Pharm. Res..

[33] Thapa N., Sharma B., Jnawali K. (2019). Expenditure for Hemodialysis: A Study among Patient Attending at Hospitals of Pokhara Metropolitan City, Nepal. J. Health Allied Sci..

[34] Endarti D., Andayani T.M., Widayanti A.W., Rohmah S., Banjarani R.R., Ghearizky N.A. (2025). Chronic disease costs from a patient’s perspective: A survey of patients with stroke, heart disease, and chronic kidney disease visiting a district hospital in Indonesia. J. Pharm. Pharmacogn. Res..

[35] Hajomer H.A., Elkhidir O.A., Elawad S.O., Elniema O.H., Khalid M.K., Altayib L.S., Abdalla I.A., Mahmoud T.A. (2024). The burden of end-stage renal disease in Khartoum, Sudan: Cost of illness study. J. Med. Econ..

[36] Li P.K.T., Lu W., Mak S.K., Boudville N., Yu X., Wu M.J., Cheng Y.L., Chan C.T., Goh B.L., Tian N. (2022). Peritoneal dialysis first policy in Hong Kong for 35 years: Global impact. Nephrology.

[37] Giuliani A., Karopadi A.N., Prieto-Velasco M., Manani S.M., Crepaldi C., Ronco C. (2017). Worldwide experiences with assisted peritoneal dialysis. Perit. Dial. Int..

[38] Karopadi A.N., Mason G., Rettore E., Ronco C. (2013). Cost of peritoneal dialysis and haemodialysis across the world. Nephrol. Dial. Transplant..

[39] Sangthawan P., Klyprayong P., Geater S.L., Tanvejsilp P., Anutrakulchai S., Boongird S., Gojaseni P., Kuhiran C., Lorvinitnun P., Noppakun K. (2022). The hidden financial catastrophe of chronic kidney disease under universal coverage and Thai “Peritoneal Dialysis First Policy”. Front. Public Health.

[40] Tang C.H., Chen H.H., Wu M.J., Hsu B.G., Tsai J.C., Kuo C.C., Lin S.P., Chen T.H., Sue Y.M. (2019). Out-of-pocket costs and productivity losses in haemodialysis and peritoneal dialysis from a patient interview survey in Taiwan. BMJ Open.

[41] Zhang H., Zhang C., Zhu S., Ye H., Zhang D. (2020). Direct medical costs of end-stage kidney disease and renal replacement therapy: A cohort study in Guangzhou City, southern China. BMC Health Serv. Res..

[42] Toure A.O., Balde M.D., Diallo A., Camara S., Soumah A.M., Sall A.O., Kourouma K., Camara B.S., Bocoum F.Y., Kouanda S. (2022). The direct cost of dialysis supported by families for patients with chronic renal failure in Ouagadougou (Burkina Faso). BMC Nephrol..

[43] NHS England (2024). Dialysis Transport Support Offer.

